# Semi-automated object-based image analysis for peri-urban land use/cover classification using WorldView-3 imagery of Mankweng, South Africa

**DOI:** 10.1038/s41598-026-66042-x

**Published:** 2026-08-28

**Authors:** Deepthi Patric, Martin Kappas, Daniel Wyss, Gertrud Schaab

**Affiliations:** 1 Department of Cartography, GIS and Remote Sensing, University of Göttingen, 37077 Göttingen, Germany; 2 Institute of Applied Research, Karlsruhe University of Applied Sciences, 76133 Karlsruhe, Germany

**Keywords:** Very high-resolution satellite imagery, WorldView-3, Former homeland area, OBIA feature selection, Fine-grained land use/cover, Ecology, Ecology, Environmental sciences, Environmental social sciences, Mathematics and computing

## Abstract

Peri-urban landscapes in semi-arid regions exhibit high spatial heterogeneity and fragmented land use/cover (LUC) patterns, challenging traditional classification methods. This study introduces a semi-automated, hybrid Object-Based Image Analysis (OBIA) framework for detailed LUC classification using WorldView-3 imagery of Mankweng, South Africa. The novelty of the approach lies in the sequential, order-dependent integration of statistical feature selection methods, specifically Coefficient of Variation (CV), Feature Space Optimization (FSO), Linear Discriminant Analysis (LDA), and Otsu’s Multi-Thresholding, where each method resolves a limitation left by the previous step rather than being applied independently. These methods reduce the trial-and-error process in feature selection and rule-set development, enabling finer class separation. The Soil Adjusted Vegetation Index (SAVI) and New Built-up Extraction Index (NBEI), used as spectral indices, and Canny edge detection, used as an image derivative layer, were embedded as supplementary input layers to improve object segmentation across hierarchical levels. A four-level Hierarchical Classification Scheme (HCS) guided segmentation and classification, enabling the differentiation of 38 LUC subclasses, including roof types and diverse vegetation types. Applied to four peri-urban test sites within the study area, the method indirectly revealed settlement structures aligned with varying socio-economic and development stages. Compared to standard OBIA, the workflow improves classification efficiency while maintaining thematic detail without relying on extensive training data. The framework provides transferable rulesets adaptable to other fragmented, data-scarce peri-urban environments. It demonstrates that combining statistical measures with known OBIA-features, reduces user input, supporting a semi-automated approach that is helpful for spatial image analysis and ultimately policy-relevant urban planning.

**Supplementary Information:**

The online version contains supplementary material available at https://doi.org/10.1038/s41598-026-66042-x.

## Introduction

Mapping land use/cover (LUC) in peri-urban areas is increasingly important for understanding spatial transformations, infrastructure planning, and resource management in rapidly evolving landscapes^1^. However, the spatial and thematic complexity of peri-urban environments presents substantial challenges for traditional classification techniques. Peri-urban zones are transitional areas between rural and urban land uses^2^. They are typically characterized by fragmented land patterns, high spatial heterogeneity, and unclear boundaries. In the African context, such zones often lack a clear urban–rural gradient and instead exhibit a patchwork of informal and formal settlements, agricultural plots, institutional infrastructure, and diverse vegetation types^3^. Mankweng and surroundings, located in Limpopo Province, South Africa, exemplifies these characteristics. As a former homeland settlement centered around the University of Limpopo, it has evolved under post-apartheid governance and mixed socio-economic conditions^4^. In this context of uneven transformations in Mankweng^5^, accurate mapping of LUC is crucial for understanding livelihood transitions and addressing infrastructure disparities driven by socio-economic fragmentation.

Despite growing interest in peri-urban studies, Sahana et al.^6^ highlight the absence of a standardized method for delineating these zones, further complicating classification efforts. Blaschke^7^ reviewed the limitations of traditional per-pixel classification methods and demonstrated that these approaches are less effective in complex environments when compared to Object-Based Image Analysis (OBIA) techniques. It has been shown that pixel-by-pixel classification methods are poorly suited for peri-urban environments due to the complex nature of the landscape, including spatial heterogeneity, fragmented land cover, and mixed land use patterns^8^. They struggle to capture the shape, context, and hierarchy of landscape features, particularly in areas without clear gradients or with fine-scale heterogeneity^9^.

Compared to pixel-based approaches, Object-Based Image Analysis (OBIA) has proven more effective in complex and heterogeneous landscapes, particularly when using very high-resolution imagery such as WorldView-3^10^. OBIA segments imagery into meaningful objects based on both spectral and spatial features, enabling the inclusion of shape, texture, and contextual information^7,11^. OBIA-based classifications, such as those proposed by Mathan and Krishnaveni^12^, focus on detecting general patterns but often fail to resolve fine-grained LUC classes or capture the fragmentation typical of peri-urban settings. Other studies, including Gao et al.^13^ and Smollich^1^, attempt to delineate the boundaries of built-up structures for humanitarian applications and peri-urban analysis. While the number of classes in these studies is adequate for their purposes, they are generally limited in both thematic detail and spatial applicability, often focusing on more homogeneous landscapes.

OBIA typically consists of two phases: (1) image segmentation and (2) feature extraction and classification. Segmentation is a critical step, as the accuracy of classification depends on the quality of the generated image objects^14–16^. Nevertheless, segmentation scale selection remains a significant limitation, especially in spatially non-uniform regions^17^. Additionally, OBIA studies often classify only broad land categories and neglect the finer LUC distinctions required for peri-urban analysis^18^. To address these limitations, there is growing interest in semi-automated or hybrid OBIA workflows that reduce user input and enhance classification consistency^19^.

In this work, we present a semi-automated OBIA framework applied to the peri-urban landscape of Mankweng and its surroundings. The novelty of this framework lies in the sequential integration of CV, FSO, LDA, and Otsu thresholding into a single ordered pipeline, an integration that has not been previously documented in OBIA based peri-urban land use/cover classification. The approach integrates eCognition software with Python scripting and incorporates a suite of statistical measures beyond their conventional binary applications, specifically to reduce the trial-and-error space in feature selection and ruleset development. By embedding these tools within the OBIA workflow, the framework minimizes reliance on manual rule-setting and iterative parameter tuning^20,21^. Rule-set development nonetheless remained strongly grounded in local area knowledge, including field-based familiarity with settlement typologies, roofing materials, and vegetation patterns characteristic of the Mankweng study area, so that statistical outputs were consistently interpreted within a locally informed classification logic. It enables the classification of a wide range of distinct LUC classes across various land use/cover categories and improves thematic resolution in spatially heterogeneous peri-urban environments. The integration of computational tools with structured rulesets enhances classification consistency for revealing spatial and socio-economic analysis in a peri-urban setting.

## Study area

**Fig. 1:**
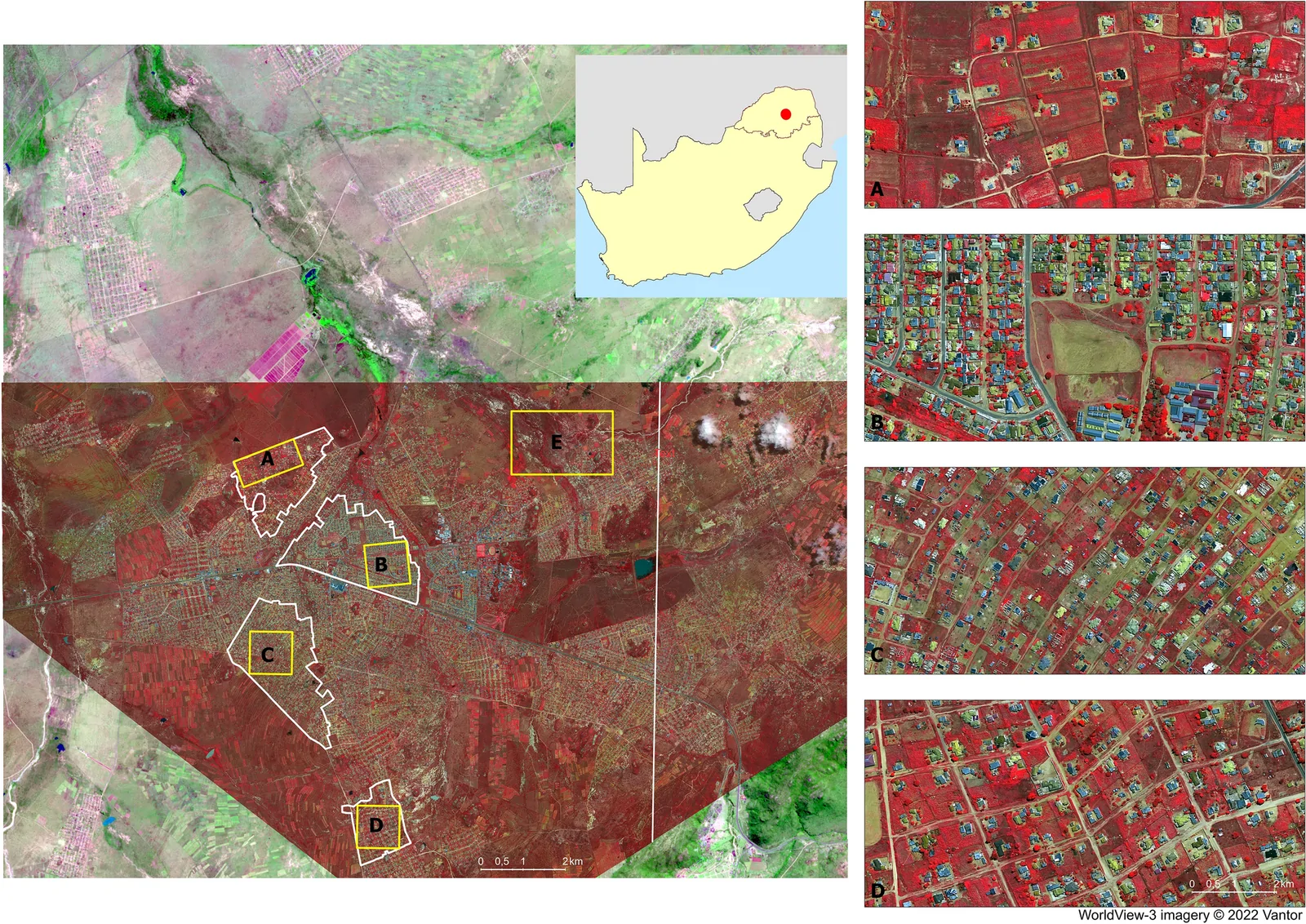
The study area is Mankweng (− 23.90 latitude, 29.72 longitude) and its environs, a peri-urban region in South Africa’s Limpopo Province, located approximately 40 km east of Polokwane. The total area covers 400 km^2^, shown in Sentinel-2 imagery (11 April 2021; bands 10, 7, 2), with analysis focused on 200 km^2^ of WorldView-3 imagery (20 March 2022; bands 7, 5, 3). Four primary study sites, outlined in white, were selected as part of the WoPedyP project. The selected subsets for testing the process, marked in yellow, are: A (Nchechane), B (Mankweng), C (Nobody), D (Ramogale), and an additional subset, Site E. The right panel shows zoomed-in views of Sites A, B, C, and D to highlight site-specific differences. Apparent visual differences reflect variation in land use density across sites. Subset E (3.75 km^2^) was included solely to ensure sufficient representation of LUC classes not adequately captured within the four 1 km^2^ subsets.

The study area focuses on peri-urban landscapes in Mankweng, and its surroundings situated in the north of Limpopo province. It forms part of the Women’s Perceptions and Place Dynamics Project (WoPedyP) which focuses on women’s perspective on active place shaping of peri urban livelihoods to accelerate transitioning to more sustainable livelihoods. The overarching aim of the project is to link women’s perception to the physical space using remote sensing as a tool to subsequently develop spatially explicit scenario simulations by combining social science findings and fine-grained spatial data for an improved understanding of the situations on the ground. The total project area is 400 km^2^ and is located 40 km east of Polokwane. WorldView-3 imagery is available for 200 km^2^ forming the focus study area of this paper, UTM coordinates 767,100/7,368,100 (SE) and 787,100/7,348,100 (NW, UTM Zone 35S based on WGS84) (Fig. 1).

Four 1 km^2^ subsets were selected from the primary WoPedyP study sites: Nchechane (A), Mankweng (B), Nobody (C), and Ramogale (D). An additional subset (E, 3.75 km^2^) was included solely to ensure representation of LUC classes not adequately captured within the four main subsets, such as dry riverbed, outcrop, and certain vegetation types. The Mankweng township, situated in one of South Africa’s former homelands, developed around the University of Limpopo formerly known as the University of the North, from 1959 onwards^4^. Settlement expansion accelerated after apartheid, with peri-urban settlements extending south and north of the road (R71) that traverse the area from west to east. Rapid post-apartheid growth along major roads have resulted in diverse settlement structures with varying erratic levels of infrastructure development^22^. These differences in settlement arrangement and building diversity as well as socio-economic variations are readily visible in very high-resolution satellite imagery (see Fig. 1 to the right) with the four sites chosen meant to capture a gradient useful for understanding the peri-urban pattern in Mankweng and its surroundings. Here, settlement expansion has led to significant land use and cover changes (LUCC), with former agricultural land being converted for residential and commercial use^23^. The region’s geography consists of flat terrain with scattered rocky outcrops (“koppies”), exposed soil patches consisting of very fine sand to clay, non-perennial water bodies including streams, and various vegetation types, including grassland, bushland and protected areas such as the Turfloop Nature Reserve^24^. Agriculture plays a central role, supplemented by communal farming (“mashemo”), maize and a variety of seasonal crops^25^. The area has a semi-arid climate (BSk: semi-arid steppe climate, Köppen classification;^26^), with peak precipitation occurring between October and March^27^.

Ground-truthing efforts in 2022 and 2023 highlighted the heterogeneity and highly dynamic nature of settlement development at various stages of establishment and regularization^28^. Governance in Mankweng and its surroundings combine traditional and municipal structures, shaping socio-economic conditions, land use patterns, and infrastructure expansion^29^. Over time, it has transitioned from a university-centered settlement into a peri-urban landscape influenced by migration and socio-economic dynamics of land tenure changes^30^. Economic activities include formal and informal employment, and small-scale trade, with key amenities such as schools, healthcare centers, and commercial zones contributing to ongoing urbanization^22^. Mankweng’s development reflects different phases of peri-urbanization, with formal housing, less-formal settlements, and government-subsidized housing known as Reconstruction and Development Programme (RDPs) coexisting within the study area^31^. The involved socio-economic processes make Mankweng and its surroundings a representative case for examining how the heterogeneity of peri-urban landscapes can be captured through object-based image analysis (OBIA) using very high-resolution satellite imagery.

## Methodology

The main dataset for this study consists of very high-resolution satellite imagery acquired from WorldView-3 on March 20 2022. The imagery covers 200 km^2^ of Mankweng’s and its environs’ peri-urban landscape in two scenes, referred to as west and east scenes throughout the study (Fig. 1). The WorldView-3 data includes eight multispectral bands ranging from 400 nm (Coastal Blue) to 1040 nm (NIR 2) with a spatial resolution of 1.24 m, along with a panchromatic band (450–800 nm) at 0.31 m resolution^32^. The imagery has a radiometric resolution of 16-bit depth, with an overall cloud cover plus cloud shadow of approximately 4%. The east and west scenes exhibit discernible differences in intensity, hue, and saturation, with a satellite azimuth difference of approximately 20° due to an acquisition time difference of 18 s. A 4.6° difference in obliquity introduced geometric discrepancies, resulting in a seamline distortion of 12 cm prior to preprocessing. To mitigate these discrepancies, we applied orthorectification using the ALOS PALSAR RTC digital elevation model of 12.5 m resolution^33^. Preprocessing reduced pixel mismatch to 7 cm between the two scenes.

To select the optimal pan-sharpening algorithm, three methods-High Pass Filter (HPF), NNDiffuse Resolution Merge, and Modified Intensity-Hue-Saturation (IHS)-were evaluated based on previous literature^34,35^. The selected subsets were used to assess their ability to maintain the spectral fidelity while enhancing the spatial resolution of all eight multispectral bands to 0.31 m resolution of the panchromatic band. Visual and quantitative assessments identified spectral distortions and artifacts for each method, with the HPF algorithm showing superior performance in preserving edge clarity and minimizing spectral distortions^36,37^. To assess the spectral preservation capability of the selected High Pass Filter (HPF) pan-sharpening framework, an objective evaluation was carried out using the Spectral Angle Mapper (SAM) metric^38^. The evaluation was performed across a representative subset image matrix (3506 × 3321 pixels) containing 11,643,426 valid pixels covering complex, mixed peri-urban targets.

**Fig. 2:**
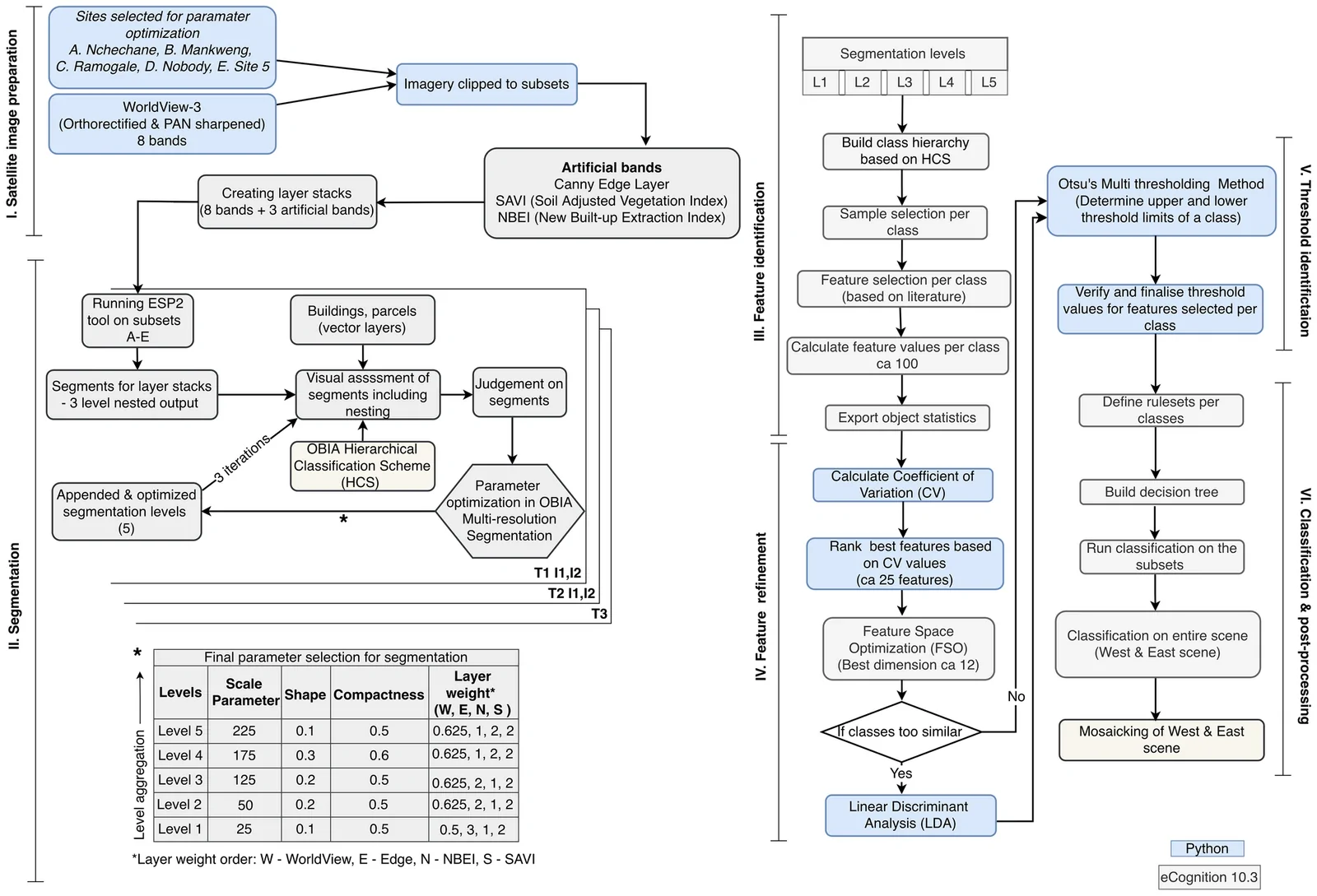
Complete and systematic workflow of the process, showing the integration of tool selection and statistical evaluations such as coefficient of variation (CV), Linear Discriminant Analysis (LDA), Otsu’s Multi Thresholding and Feature Space Optimization (FSO) are marked in each step. The entire process is categorized into six key steps, marked within the figure as I through VI.The final Scale Parameter, Shape, and Compactness values selected for Multiresolution Segmentation at each of the four hierarchical levels are indicated within Step II.

The entire process of segmentation and classification, including image preparation steps, is structured into six key steps: I. Satellite Image Preparation II. Segmentation III. Feature Identification & Ruleset Defining IV. Feature Refinement V. Threshold Identification VI. Classification & Post-Processing. (Fig. 2). The processes were first carried out within selected subsets to reduce computational load and address eCognition software limitations, enabling classification training, visual refinement of ruleset parameters, and iterative workflow testing prior to application across the full study area^39^.

Several software applications were used for image processing, segmentation, classification, and analysis. ArcGIS Pro 3.2.0 and ERDAS IMAGINE 2022 (version 16.7) were used for preprocessing, including orthorectification and pan-sharpening^40^. eCognition 10.3 was used for segmentation and classification^17^, while Python 3 packages facilitated feature selection, elimination and refinement^41^. Three supplementary layers were integrated into the dataset to enhance segmentation and classification accuracy: I. Soil Adjusted Vegetation Index (SAVI), a spectral index used to distinguish vegetation from non-vegetation^42^, calculated based on the NIR1 and Red bands with an L constant of 0.5. II. New Built-up Extraction Index (NBEI), a spectral index targeting built-up areas and discriminating roof types^43^, calculated using the NIR2, NIR1, Green, and Rededge bands. III. Canny Edge Detection, an image derivative layer applied using a 5 × 5 Gaussian filter with lower and upper thresholds of 100 and 200^44^. A Sobel Edge Filter was incorporated within the Canny Edge Detection computation to prevent edge exaggeration^45^. These supplementary layers were stacked together with the multispectral bands for segmentation, aiming to treat objects of different sizes across hierarchical levels^46^. Following the layer stack preparation, the stack combinations were iteratively assessed, with each process informing and refining the other through repeated trial runs rather than following a fixed sequential or conditional branching logic.

Based on prior ground-truthing in the study area^28^, a Hierarchical Classification Scheme (HCS) was designed to categorize land use/cover into five broad parent classes: bare ground, vegetation, infrastructure & services, water, and tarmac road. Unlike the other four parent classes, tarmac road is treated as a standalone class and is not subdivided into sub-objects. The HCS follows a bottom-up segmentation logic reflecting the region-growing approach in eCognition, where fine-scale seed objects at the finest level (L1) are progressively merged into larger objects at coarser levels^47,48^. Classification proceeds top-down approach where broad parent classes are first identified at the coarsest level and image objects are then progressively assigned to more specific subclasses at finer levels. The full HCS, including all subclasses and their assigned segmentation levels, is presented in Sect. “Hierarchical classification scheme”. Among the available segmentation algorithms in eCognition, Multiresolution Segmentation (MRS) was selected due to its effectiveness in handling heterogeneous landscapes and its parameter flexibility^49,50^. The nesting approach provided defines the nested relationships between super objects and sub objects within the multi-hierarchical approach. To refine segmentation, Multi-Threshold Segmentation (MTS) was applied in conjunction with MRS^51^, specifically the NBEI band at Level 2 was used to differentiate roof types, ensuring that Level 2 segments were further subdivided based on thresholds while maintaining the hierarchical nesting.

The ESP2 tool, based on the concept of local variance^46,52^, provided initial guidance for identifying suitable Scale Parameter (SP) values and seed segments, with the average across subsets selected as the working value. Shape and Compactness values were then optimized through iterative trials (T1, T2, T3).^46,52^. The most important parameter in MRS is the SP, which controls the average image object size and heterogeneity^46^. Two segmentation approaches were then done within the trials T1 and T2: (a) constant Shape and Compactness values (0.1 and 0.5), (b) gradual increment of Shape and Compactness values by 0.1. Throughout these trials, segments were visually assessed based on the following criteria along with the auxiliary data: (a) Region uniformity within specific classes; (b) Object coarseness and smoothness; (c) Spectral differences among neighboring segments; (d) Mean segment values within the same class; (e) Accuracy of boundary alignment with imagery features; (f) Inter-segmentation relationships, particularly nesting within the HCS framework^49,53^.

Layer weights in the Multiresolution Segmentation (MRS) algorithm were assigned according to the thematic priorities defined within the Hierarchical Classification Scheme (HCS) for each segmentation level^54^. The weighting strategy followed a structured, knowledge-based rationale in which layers were prioritised based on their perceived ability to distinguish the dominant target classes at a given hierarchical level, rather than through automated optimisation procedures. The multispectral bands consistently received the highest weights across all levels to preserve spectral integrity throughout the segmentation process. Supplementary layers were weighted according to their functional relevance at each stage of segmentation. At Level 2, the NBEI layer was prioritised to enhance discrimination between roof types and built-up surfaces. At Level 3, SAVI received greater weighting to improve separation between vegetation and non-vegetation classes. At Levels 1 and 2, the Edge layer received increased weighting to support the extraction of fine-scale features such as roof boundaries, dirt roads, and other small structural elements. To maintain balanced layer contribution and allow comparison across segmentation levels, all weights were rescaled to a relative total of 10. The complete weighting scheme and corresponding rescaled values for each segmentation level are presented in Appendix (Table S2). A closer examination of segmentation at Level 5 revealed overgeneralization of LUC classes, leading to its eventual exclusion from the workflow. The final Scale Parameter, Shape, and Compactness values selected for segmentation of the entire scene (East and West) are presented in Fig. 2.

The sample selection step in OBIA is critical due to the method’s strong reliance on training datasets^55^. Key features were identified as reviewed by Hossain and Chen^11^ and include spectral, geometric, spatial, topological, and hierarchical characteristics^56^. Feature selection was performed by identifying optimal objects for each class and selecting representative samples at the appropriate segmentation level^57^. The initial selection of features contained around 125 features per class. A Python script was used to compute the Coefficient of Variation (CV) for each class and eliminate features with high variability. Feature values were exported directly from eCognition as comma-separated files and used as input to the Python scripts. The resulting refined feature sets were subsequently re-imported into eCognition for ruleset development and classification. The complete Python scripts are available via the repository cited in the Data Availability Statement.

**Fig. 3:**
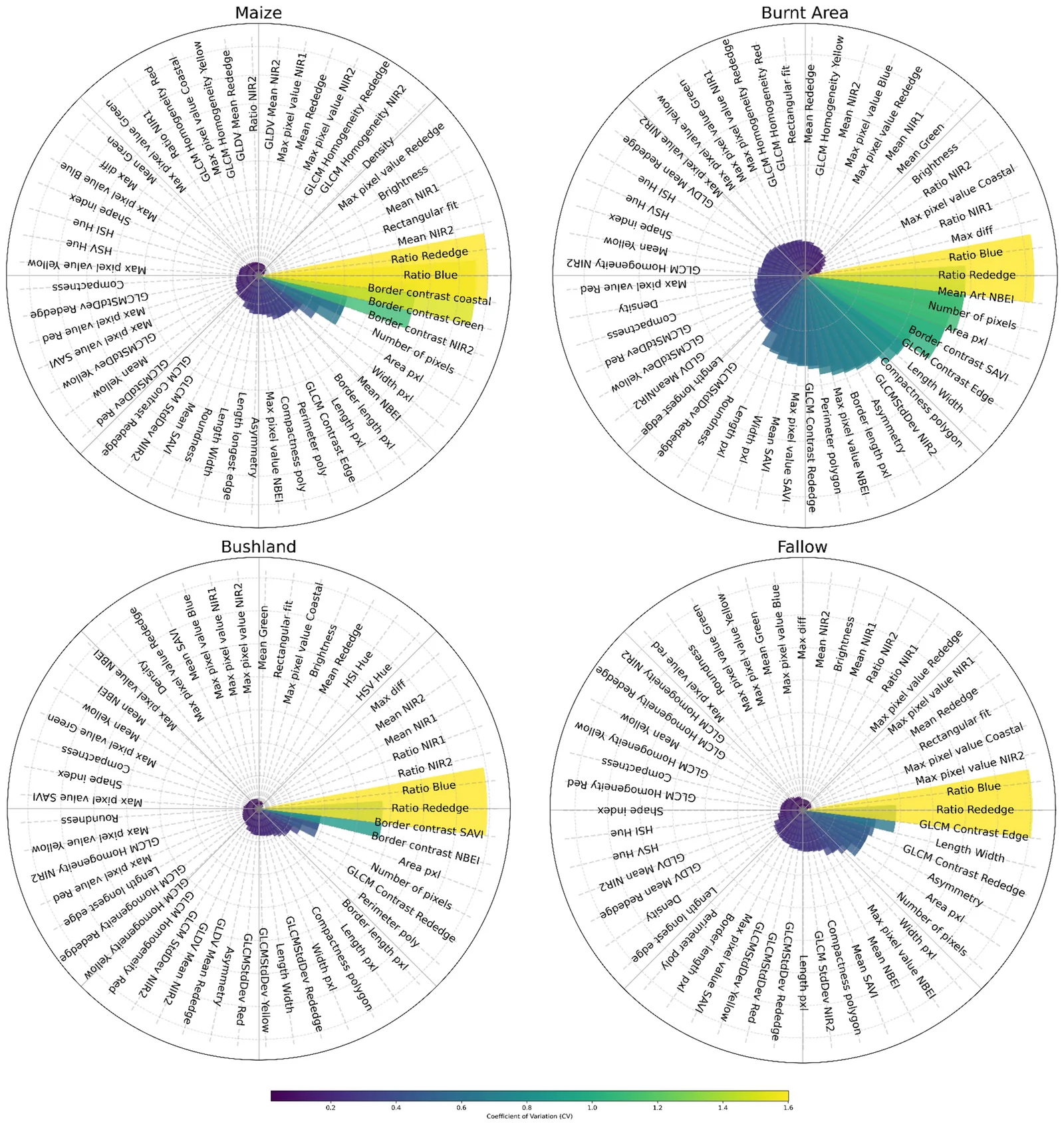
The process of feature selection involves calculating the coefficient of variation (CV) to fine tune the optimal set of features for each class. An example of comparative analysis is shown for four classes: bushland, fallow, burnt area, and maize. The radial diagrams show the initial feature selection for each class before the final selection.

CV values help in selecting the most stable features to better standardize the ranking scheme^58^. Features with a CV > 0.5 were removed to reduce background noise^59^. Features with lower CV values may be prioritized for inclusion in models, while features with higher CV values could be considered for exclusion or treated as less important^60^. For example, the ratio rededge feature for maize exhibited high variability and was excluded as an unreliable feature measure for classification. The CV is subsequently used to reduce the number of features to a maximum of 25 per class (Fig. 3).

**Fig. 4:**
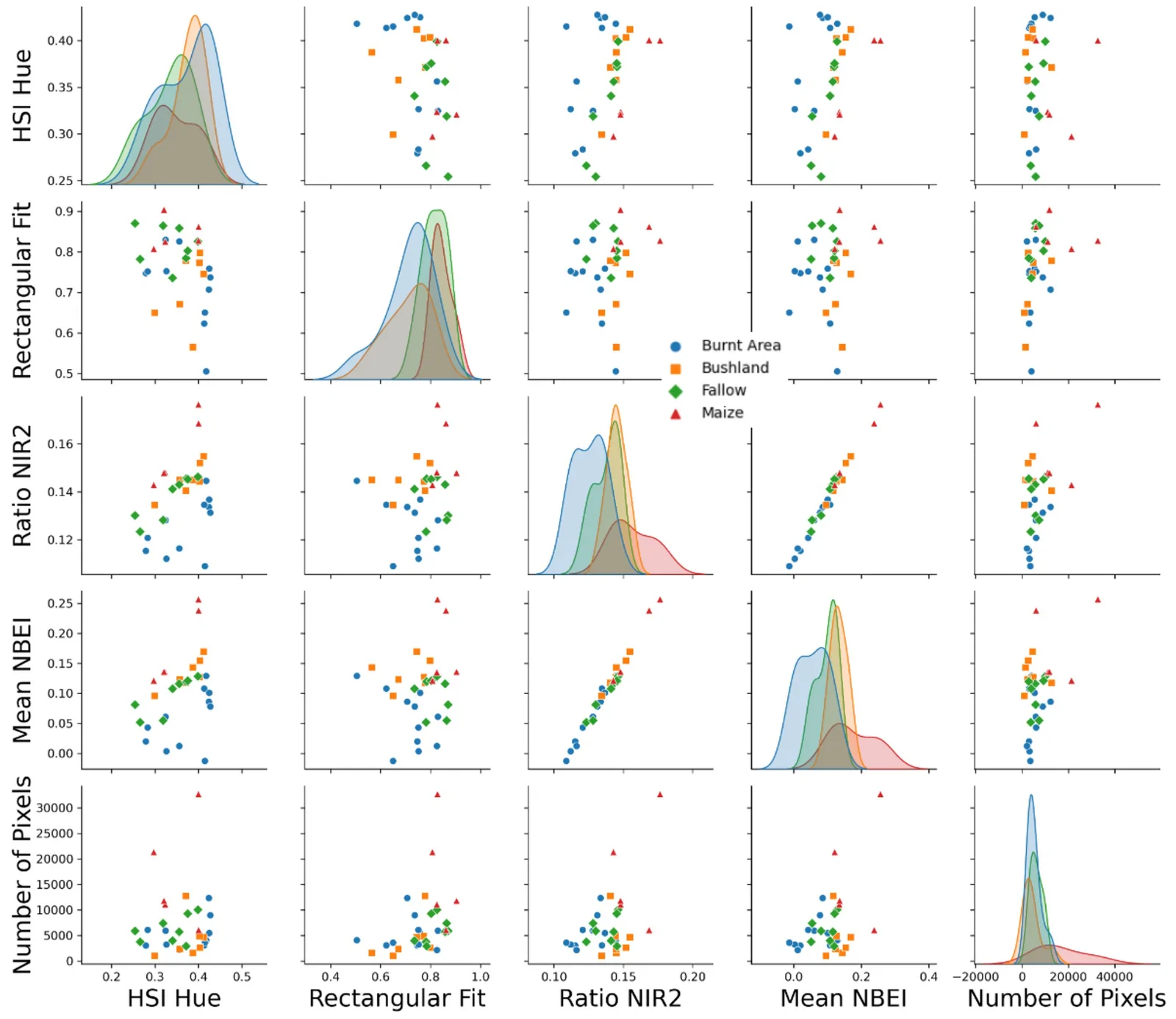
Computation of Linear Discriminant Analysis (LDA) for selected classes (maize, burnt area, bushland, and fallow) and features. The scatter plot illustrates sample distribution and overlaps between selected features within the classes, displayed as pair plots.

Next, the Feature Space Optimization (FSO) tool in eCognition was used to further refine the feature set based on class separability and dimensionality reduction^61^. It assesses Euclidean distances in the feature space among samples and identifies the combination of features that maximize class separation^62^. This process reduced the number of selected features to approximately 12 per class. To define lower and upper feature value boundaries, Otsu’s Multi-Thresholding was applied using an additional Python script, which identified optimal feature values that fall within the range of 5th and 95th percentiles, removing outliers. In special cases where classes exhibited spectral similarity, Linear Discriminant Analysis (LDA) was applied to improve class separability^63^, resolving overlaps between spectrally similar classes such as burnt area, bushland, fallow and maize. LDA identified linear combinations of features that maximised the ratio of between-class variance to within-class variance, producing discriminant boundaries subsequently used to refine feature selection and ruleset thresholds in eCognition. The pair plot (Fig. 4) illustrates the relationships between five selected features (e.g., HSI Hue, Rectangular Fit, Ratio NIR2, Mean NBEI, and Number of Pixels) across these classes. The diagonal histograms display the distribution of each feature across the four classes. Feature plots that exhibit significant overlaps between class distributions indicate limited discriminative power and overlapping density curves suggest that these features may not effectively distinguish between classes^64^. The final number of features selected for classes is presented in Table 1. The longer list of features used in the ruleset for classification is provided in the appendix as Table S1.

**Table 1:** The table lists parent classes & subclasses grouped under five parent classes: vegetation, infrastructure & services, bare ground, water, and tarmac road. The table provides the segmentation level, number of training samples used for feature selection, number of features used in ruleset development, a visual evaluation score (1: low–10: high) indicating classification accuracy, and if the subset values as derived for the Western scene could be also used for the Eastern scene.

ID	Class name	Level of segmentation	Number of samples	Number of features	Classification evaluation (1–10)	Subset values reused
1	Bare ground	4	124	6	8	Y
1.1	Bare ground ibs	3	145	5	8	Y
1.2	Sports ground	3	16	2	8	Y
1.3	Bare ground ins	3	132	5	7	Y
1.4	Dry riverbed	3	12	9	7	Y
1.5	Outcrop	3	77	11	8	Y
1.6	Erosion	3	9	3	6	N
1.7	Dumpsite	3	19	9	6	Y
1.8	Kraal	2	7	6	6	Y
1.9	Mining	3	11	2	8	N
2	Water	4	4	2	10	Y
2.1	Open water	4	12	3	9	Y
2.2	Swimming pool	4	2	3	7	Y
2.3	River	4	9	3	8	Y
3	Vegetation	4	9	4	9	Y
3.1	Maize	3	198	12	7	Y
3.2	Mashemo	3	17	5	7	Y
3.3	Fallow	3	106	12	6	Y
3.4	Bushland/Shrubland	3	134	11	6	N
3.5	Grassland/Savannah	3	122	7	6	N
3.6	Burnt area	3	5	10	3	N
3.7	Riverine vegetation	3	34	4	7	N
3.8.1	Trees	NBEI_MTS (2)	112	1	7	Y
3.8.2	Single tree	NBEI_MTS (2)	123	2	7	Y
3.8.2	Fruit tree	NBEI_MTS (2)	25	5	7	Y
3.8.3	Tree stand	NBEI_MTS (2)	78	1	7	Y
3.9	Aloe	2	46	4	3	N
4	Built-up & infrastructure	–				
4.1	Tarmac road	4	13	6	8	Y
4.2.1	Metal roof	NBEI_MTS (2)	167	1	8	Y
4.2.2	Slate roof	NBEI_MTS (2)	167	1	8	Y
4.2.3	Thatched roof	NBEI_MTS (2)	12	1	7	Y
4.2.4	Unfinished roof	NBEI_MTS (2)	112	1	6	Y
4.3	Commercial buildings	3	3	3	8	Y
4.4	School	3	3	2	8	Y
4.5	Sheds	NBEI_MTS (2)	77	2	6	Y
4.6	RDP house	NBEI_MTS (2)	68	2	6	Y
4.7	Wall/Fence	2	109	1	4	N
4.8	Footpath	2	33	2	5	N
4.9	Dirt road	3	79	11	8	Y
4.10	Water tank closed	NBEI_MTS (2)	24	1	8	Y
4.11	Cemetery	3	41	7	8	Y
4.12	Carpark	3	13	2	7	Y

Classification accuracy was assessed using two complementary approaches. A quantitative assessment was conducted using 471 field-collected ground truth points acquired during field campaigns in 2022 and 2023^28^. Ground truth points were spatially matched to the classification layer using a 50 m buffer to account for GPS positional uncertainty, the landscape-scale nature of field observations, and minimum mapping unit constraints^65^. A point was considered correctly classified if any polygon intersecting the buffer corresponded to any LUC class recorded for that location across all field observation columns. Overall Accuracy (OA) was calculated as OA = (C / N) × 100, where C is the number of correctly classified reference points and N is the total number of reference points. The Kappa coefficient (κ) was additionally estimated to evaluate agreement beyond chance^66,67^.

For subclasses lacking sufficient ground truth coverage were evaluated visually based on spatial coherence, thematic consistency, and interpretation knowledge obtained during field verification. Visual evaluation scores ranging from 1 to 10 were assigned to each class, as presented in Table 1. Together, the quantitative and visual assessments provided a complementary framework for evaluating classification reliability across all 38 LUC subclasses.

## Results

### Hierarchical classification scheme

**Fig. 5:**
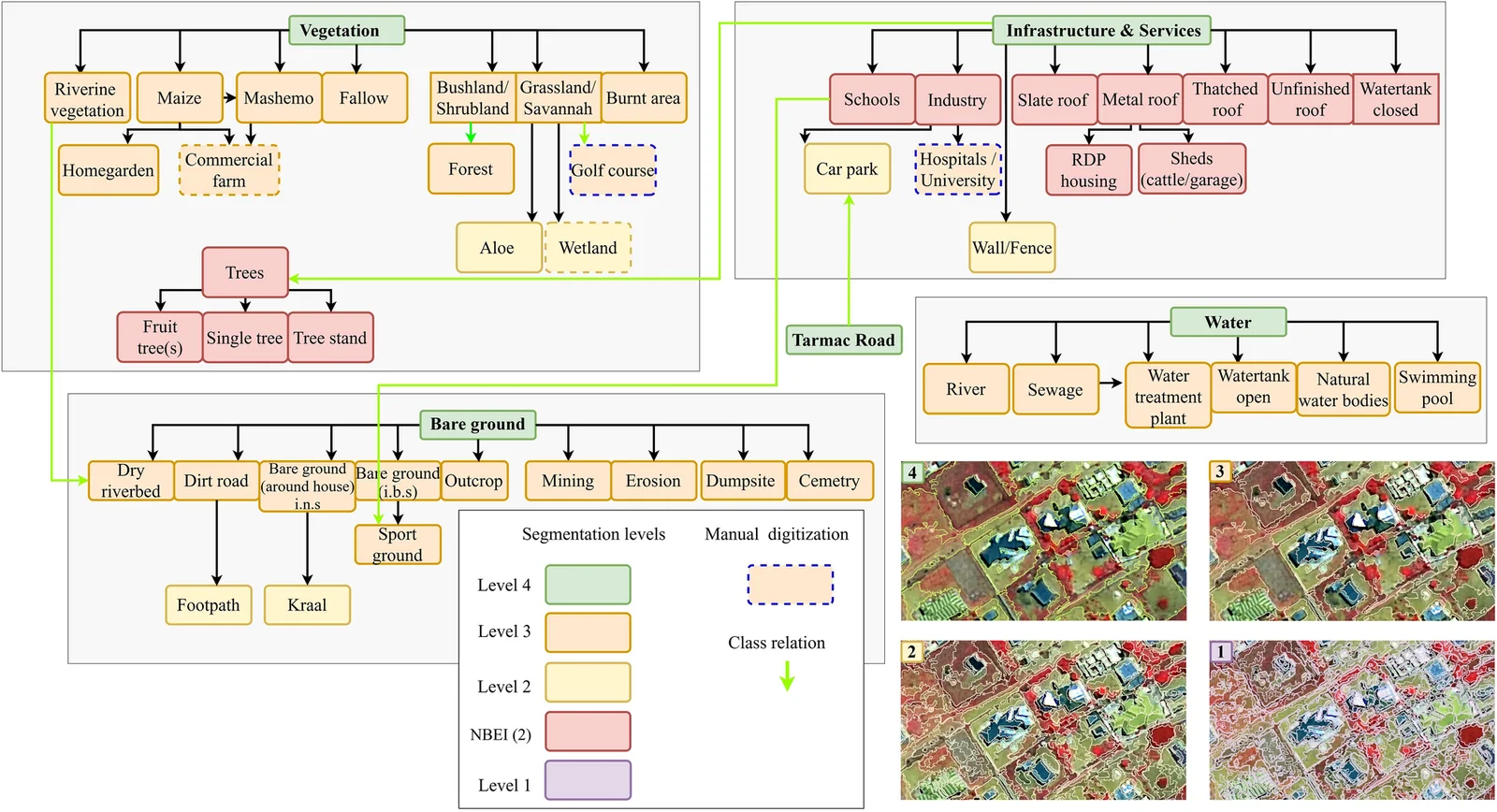
Hierarchical Classification Scheme (HCS) outlining the multi-level, nested categorization of land use/cover (LUC) classes across five parent classes: Vegetation, Infrastructure and Services, Bare Ground, Water, and Tarmac Road. Each parent class is subdivided into subclasses assigned to specific segmentation levels, colour-coded as follows: Level 4 (green), Level 3 (orange), Level 2 (yellow), NBEI2 (red, a sub-segmentation step within Level 2 for roof type and tree type differentiation). Classification proceeds top-down from coarser parent classes to finer subclasses, while segmentation follows a bottom-up region-growing logic from Level 1 upward. Dashed boxes indicate classes delineated through manual digitization. Green arrows denote cross parent–child class relations. The four imagery panels on the right illustrate object boundaries at each segmentation level, from coarsest (Level 4) to finest (Level 1).

The final Hierarchical Classification Scheme (HCS) comprises land use/cover (LUC) subclasses organized within five parent classes and four segmentation levels, reflecting the thematic and spatial complexity of the peri-urban landscape in Mankweng (Fig. 5).

The classification system follows a structured, four-level hierarchy that reflects the nested, tree-like structure of OBIA. It groups classes into parent and child relationships across segmentation object levels,complexity in the peri-urban landscape of Mankweng. The primary classes include four broad categories: vegetation, infrastructure & services, water, bare ground plus tarmac road at Level 4, which are progressively subdivided into more specific subclasses based on spatial, spectral, and contextual attributes. Each of these parent classes is subdivided into progressively finer subclasses through Levels 3, 2 & 1, reflecting increasing levels of spatial and semantic detail. For example, within the vegetation class (Level 4), Level 3 subclasses include categories such as bushland/shrubland, grassland/savannah, and commercial farm. These are further subdivided at Level 2 into more specific units like aloe, and wetland. At the detailed Level 2, object types such as fruit tree(s), tree stand, and single tree are distinguished under the general trees category. This distinction carries potential socio-economic relevance: fruit trees around homesteads may indicate more established households and reduced dependence on purely subsistence-based agricultural practices, whereas single trees and tree stands are more commonly associated with grazing areas and transitional bushland environments. Separating these subclasses allows for a more nuanced representation of vegetation structure within the classification and may support exploratory interpretation of livelihood-related landscape patterns and settlement development stages, in line with the applied objectives of this study. Similarly, the infrastructure & services class at Level 4 is refined into subclasses such as various roof types, based on the building material types.

At segmentation level 2, the Multi-Resolution Segmentation (MRS) derived segments were further subdivided using the Multi-Threshold Segmentation (MTS) algorithm, while maintaining their nested structure. This was done using the NBEI supplementary layer, with a specific focus on distinguishing building roof types. The resulting thresholds were then applied to classify four distinct roof types (e.g., metal roof, thatched roof, unfinished roof and slate roof), utility structures (e.g., water tank closed), and institutional buildings (e.g., schools, industry, hospitals/university). The water class branches into subclasses like river, sewage, natural water bodies, and swimming pool, while the bare ground class includes dry riverbed, dirt road, mining, and outcrop, among others. The tarmac road class is treated as an independent, non-branching land use/cover category. Table 1 lists all subclasses distinguished in the classification scheme and provides, for each, a landscape-based definition and the assigned segmentation level. Level 4 includes five parent classes. Level 3 contains 28 subclasses, comprising 11 under vegetation, one under infrastructure & services, 10 under bare ground, and six under water. Level 2, including those derived from supplementary MTS thresholds, contains 19 subclasses: six under Vegetation, 11 under infrastructure & services, and two under bare ground. Figure 5 summarises the class hierarchy and representative segmentation outputs across the four hierarchical levels. Broader classes (e.g. vegetation, bare ground, infrastructure & services) are captured at higher levels, while Levels 2 and 3 refine these into more specific classes (e.g. roof types, trees). These levels correspond directly to segmentation object granularity in OBIA.

Spatial, spectral, and contextual rules are noted for each subclass where relevant. Contextual dependencies are used for classes such as riverine vegetation and sports ground, which are classified partly based on proximity to linear features like rivers or adjacent land use types such as schools. Spectral and textural distinctions, such as those used to identify burnt area and outcrop, are also listed. The contextual dependencies as well as the inter dependence of classes in various hierarchical levels in the classification are marked in Fig. 5 with arrows.

### LUC classifications of the study sites

**Fig. 6:**
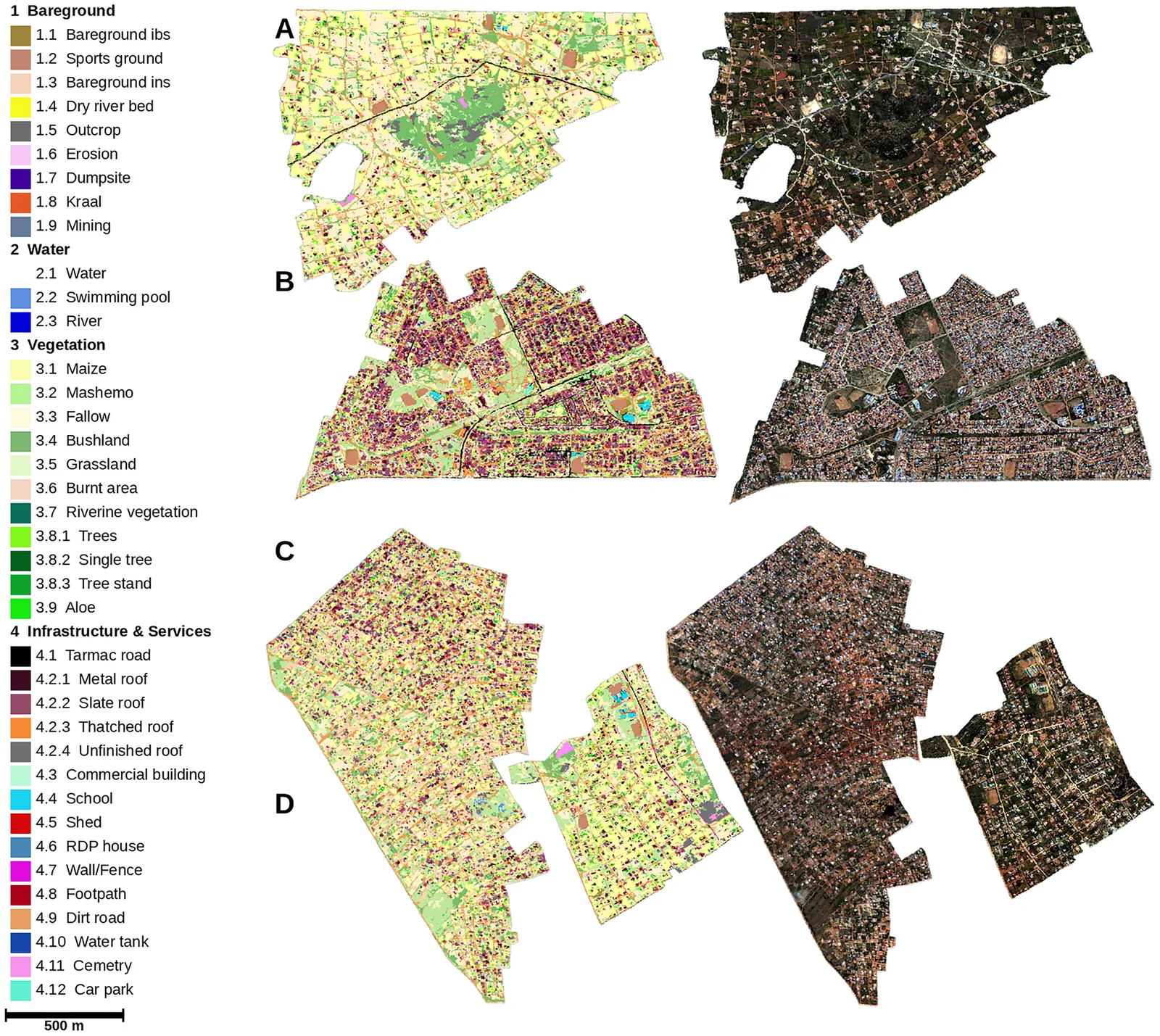
Final LUC classification maps (centre) and corresponding raw WorldView-3 imagery in natural colour composite (right) for the four study sites: **A** (Nchechane), **B** (Mankweng), **C** (Nobody), and **D** (Ramogale).

The final classified LUC maps for the four WoPedyP study sites: Nchechane (A), Mankweng (B), Nobody(C), and Ramogale (D) are shown in Fig. 6. The resulting maps highlight spatially explicit differences in land use/cover composition across the four sites. Overall, they reflect a heterogeneous mix of land use types, activities, and stages of peri-urban development characteristic of the study area. The spatial arrangement and interfaces between different land use categories become particularly apparent at finer inspection, revealing variations in settlement structure, vegetation distribution, and built-up patterns across sites.

Mankweng displays a regular gridded built-up pattern with large slate-roofed structures, planned infrastructure, and open spaces such as sports grounds. Nchechane is characterised by large stand sizes with predominantly metal sheds, extensive maize and fallow land, and a higher presence of kraals indicating livestock as a form of livelihood. Nobody shows a heterogeneous mix of settlement types at various stages of development, with unfinished roofs and dense dirt road networks. Ramogale presents the most regular settlement pattern, with uniformly distributed dirt roads, medium-sized houses, and maize fields within stands. A more distinct pattern of densification is visible in the northern part of the site compared to the south. The maps visually represent the spatial distribution of land use and cover types across each area, clearly delineating broad and finer classes as per the classification scheme discussed in Sect. “Hierarchical classification scheme”. It captures structural differences and a clear gradient between the sites needed for understanding the peri-urban heterogeneity in the region. The high granularity of the classification improves understanding of the socio-economic situation on the ground and reveals patterns of livelihood between the study sites. Fine-scale classes, such as distinct roof types and fruit trees, serve as indirect proxies of household wealth. Slate roofs are associated with higher wealth compared to small metal shacks, which often represent newer houses. Fruit trees around the houses indicate older households that are less reliant on subsistence maize farming, despite maize being the staple crop.

**Fig. 7:**
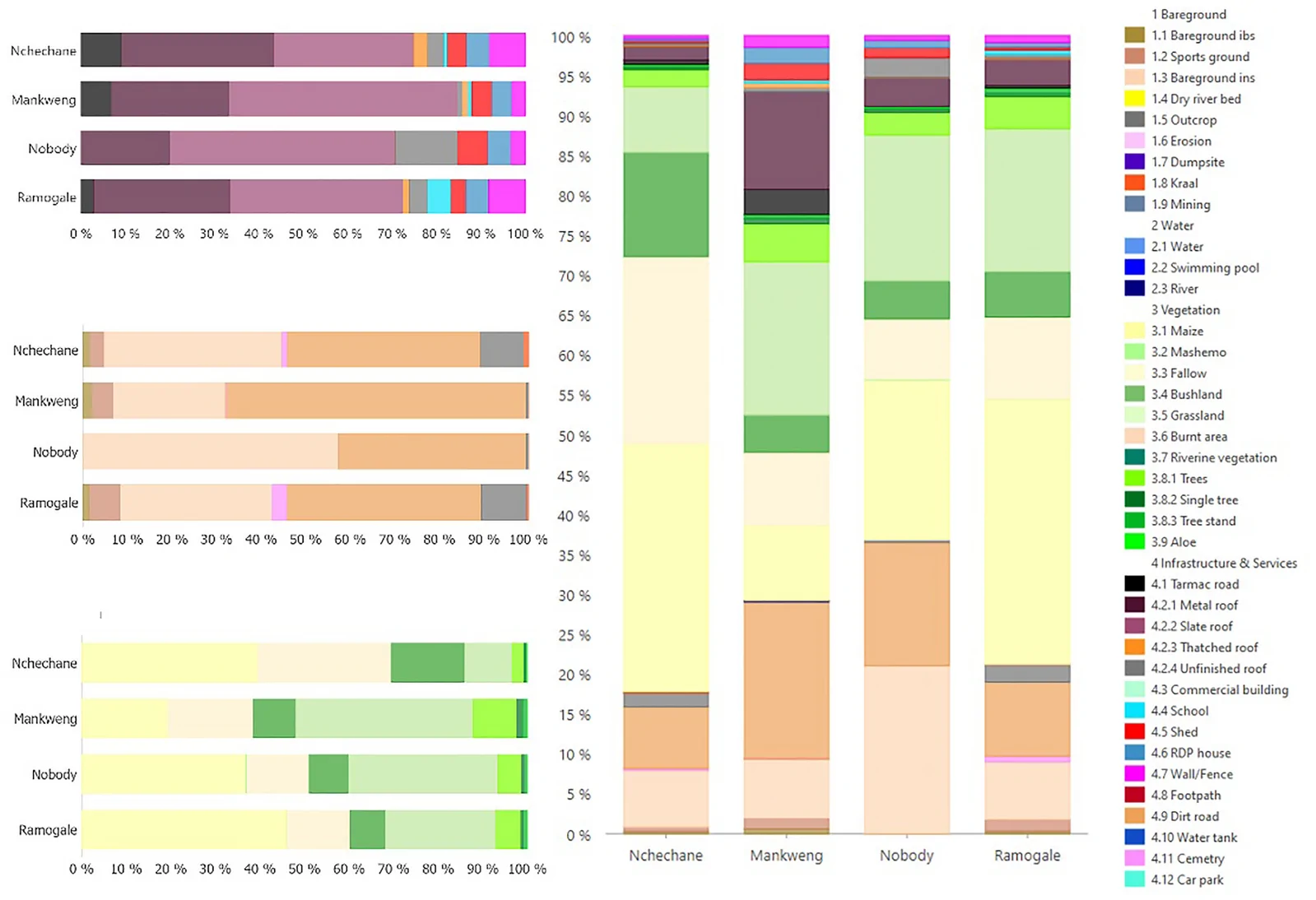
The right graph shows the overall proportion of LUC categories for each site. Each bar on the left represents the proportion of classes (%) allocated to the broader LUC classes (bare ground, vegetation, built-up & infrastructure) within the respective study sites, allowing for detailed comparison.

Figure 7 presents the proportional LUC composition for each site, grouped into three broad categories: bare ground, vegetation, and infrastructure and services. These proportions standardise class representation across sites of varying total area, enabling direct comparison of land-cover dominance and structural composition. Examining proportional LUC composition allows socioeconomic differences between sites to be inferred from the physical landscape: roof material distribution reflects housing quality and investment levels, bare ground extent indicates settlement consolidation stage, and vegetation cover patterns reveal the degree of dependence on agriculture and informal land use. The plots reveal clear inter-site differences in spatial organisation that align with varying stages of peri-urban development across the four selected study sites. In the infrastructure and services category, Mankweng is characterised by a higher proportion of structured roofed surfaces, particularly slate and metal roofs, reflecting a more consolidated built environment and higher levels of formal housing investment. In contrast, Nchechane and Ramogale show a stronger agricultural and transitional settlement character, with vegetation and bare ground classes dominating over built infrastructure, indicative of earlier development stages and greater reliance on subsistence land use. Nobody is distinguished by the highest proportion of bare ground inside settlements across all four sites, indicating a less consolidated spatial structure and ongoing informal settlement expansion. Vegetation patterns further highlight the dominance of maize-based agriculture in Nchechane and Ramogale, whereas Mankweng and Nobody exhibit more mixed vegetation cover reflecting different land use pressures. Together, these proportional distributions demonstrate that fine-grained OBIA classification can serve as an indirect but spatially explicit proxy for socioeconomic differentiation across peri-urban landscapes.

The classified maps in comparison with the LUC proportions capture distinct LUC profiles across the sites, highlighting the heterogeneity of peri-urban environments through the spatial distribution of infrastructure, agricultural areas, and natural vegetation.

### Accuracy assessment

The accuracy of the LUC classification was assessed using 471 field-collected ground truth points acquired during surveys in 2022 and 2023^28^. Of these, 205 points were excluded prior to accuracy assessment, following three explicit, programmatically applied criteria. First, points whose field-recorded label could not be reliably mapped to a class in the classification scheme were excluded,this included ambiguous or non-diagnostic descriptions (e.g. “no agriculture,” “nil”), decorative or ornamental land uses not represented as distinct classes (e.g. “garden”), and labels referring to entities outside the classification scheme (e.g. “student center,” “tap”). Points for which no classification polygon intersected the 50 m buffer around the recorded coordinate were excluded, as no predicted class was available for comparison. A small number of points fell outside the geographic bounds of the study area, likely due to GPS or transcription error during fieldwork, and were excluded accordingly. Applying these criteria in combination left 266 valid reference points spanning 13 of the 38 LUC subclasses. A point was classified as correctly predicted if the classification polygon intersecting the buffer matched any observed LUC class recorded at that location across all field data columns, reflecting the multi-class nature of peri-urban ground observations.

The classification achieved an overall accuracy of 90.2% and a Kappa coefficient of 0.81, indicating agreement between the classification and reference data^67^. Per-class accuracy results are presented in Table 2. Classes with strong thematic distinctiveness, including metal roofs, dirt roads, maize, and grassland, achieved the highest accuracies, consistent with their high visual evaluation scores and clear spectral and spatial separability. In contrast, bare ground in-settlement and dumpsites showed comparatively lower accuracies, reflecting their fragmented distribution and spectral similarity to surrounding bare-ground surfaces. Outcrop classes exhibited moderate accuracy, likely due to overlap with spectrally similar bare-ground categories.

**Table 2:** Per-class accuracy assessment results based on ground truth points. Overall Accuracy = 90.2%; estimated Kappa = 0.81.

Class name	No. of observations	Correct observations	Accuracy (%)
Bare ground (outside settlement)	13	5	38.5
Cemetery	2	2	100.0
Dirt road	21	21	100.0
Dumpsite	7	2	28.6
Fallow	1	1	100.0
Grassland/Savannah	12	12	100.0
Industry	1	0	0.0
Maize	12	12	100.0
Mashemo	1	1	100.0
Metal roof	182	175	96.2
Outcrop	9	6	66.7
Tarmac road	1	0	0.0
Single tree / Fruit tree	4	3	75.0
Overall	266	240	90.2% Kappa ≈ 0.81

The accuracy assessment remains partial because quantitative validation was only possible for a limited subclass. Inaccessible, or infrequently observed classes, including burnt area, aloe, swimming pool, dry riverbed, and wetland, could not be assessed using ground reference data and were therefore evaluated through visual interpretation. Despite these limitations, the overall accuracy of the dominant land-cover classes support subsequent interpretation of the LUC patterns presented in Sect. “LUC classifications of the study sites”.

For contextual comparison, only a few previous classification studies have been conducted in the Mankweng area, and these have primarily relied on pixel-based approaches using coarser-resolution imagery and broader class schemes. Muavhi^68^ applied supervised pixel-based classification to ASTER imagery and reported producer’s and user’s accuracies ranging from 80 to 100% across six broad land cover classes, representing a substantially lower thematic complexity than the 38 LUC subclasses distinguished in this study. More recently, Vásquez Tavera et al.^69^ used Landsat time-series analysis across the same study area and reported an overall accuracy of 61.53% with a Kappa coefficient of 0.57 for broader settlement typology classes. Although direct metric-to-metric comparison is constrained by differences in spatial resolution, class structure, and validation methodology, the 90.2% overall accuracy and Kappa coefficient of 0.81 achieved in this study suggest improved classification performance under a substantially finer thematic resolution using VHR WorldView-3 imagery and an OBIA-based framework.

For classes lacking ground truth reference data, classification performance was assessed through visual interpretation informed by field observations and local landscape knowledge. Visual evaluation scores ranging from 1 to 10 are reported in Table 1, where higher scores indicate greater confidence in class delineation and lower scores indicate increased uncertainty. Classes with strong spectral and spatial distinctiveness, such as water bodies and tarmac roads, consistently achieved high evaluation scores. In contrast, classes including burnt area, aloe, wall/fence, kraal, and dumpsite received lower scores, reflecting greater classification difficulty arising from spectral similarity, spatial fragmentation, or limited class representation.

Table 1 also reports the number of training samples used for rule-set development. Sample sizes were proportional to class representation within the selected subsets and varied according to class complexity and spectral variability. Classes such as maize and fallow land required larger training datasets to capture intra-class variability and improve separation from spectrally similar classes, whereas rare classes such as burnt area were represented by fewer samples due to their limited occurrence within the study area.

## Discussion

The aim of this study was to develop a detailed land use/cover (LUC) classification framework for the heterogeneous peri-urban environment of Mankweng and its surroundings using a semi-automated Object-Based Image Analysis (OBIA) approach. Compared to conventional pixel-based classification, OBIA incorporates spectral, spatial, contextual, and hierarchical characteristics of image objects, making it better suited for fragmented and mixed-use peri-urban landscapes^7,70,71^. Despite its advantages, OBIA workflows often remain highly dependent on manual trial-and-error due to the wide range of segmentation parameters, feature combinations, and rule-based options available^48^. Previous studies have also highlighted difficulties in systematically integrating statistical feature selection methods and supplementary input layers into operational OBIA workflows^19^. Dronova^72^, in a review of 73 OBIA studies, observed that hierarchical segmentation, adaptive parameter tuning, and landscape-informed rulesets are rarely combined within a single framework.

Although the proposed methods have individually been applied in remote sensing applications^73^, their coordinated integration within a hierarchical OBIA workflow, structured as a sequential, multi-stage pipeline, has not previously been documented (Fig. 2). Rather than following a disjointed or purely parallel application, as is typical of standard OBIA workflows that rely on user-driven, iterative trial-and-error for feature selection and threshold determination^7,72^, each stage of the proposed framework directly conditions the data for the next. CV first stabilizes the feature space by removing highly variable, unreliable parameters, reducing the initial pool of over 125 features per class to a maximum of 25. FSO then takes this stable subset and isolates the combination of features that maximizes Euclidean multi-class distance, reducing the set further to approximately 12 features. For spectrally persistent overlaps that Euclidean distance alone cannot resolve, such as separating peri-urban agricultural classes like maize from surrounding bushland, LDA calculates discriminant boundaries based on class variance ratios. Finally, Otsu’s multi-thresholding automatically derives the lower and upper ruleset boundaries within the 5th and 95th percentile range of the retained features. By formalizing this sequence, the framework reduces the manual, expert-dependent parameter tuning typical of conventional OBIA workflows to an automated procedure applied consistently across features and classes, addressing the integration gap identified by Blaschke et al.^19^ and Dronova^72^. In particular, Otsu’s method has mainly been applied in binary form within OBIA studies, whereas its extension to multi-thresholding enabled more flexible class separation within the hierarchical ruleset developed here.

At the applied level, the framework enabled the differentiation of 38 LUC subclasses representing settlement types, roofing materials, vegetation classes, and infrastructure components within a former homeland peri-urban context in sub-Saharan Africa. The combination of automated statistical procedures with expert-guided rule development reduced the extent of manual trial-and-error typically associated with OBIA while maintaining detailed thematic resolution. As such, the workflow provides a structured and potentially transferable approach for mapping highly heterogeneous and data-scarce peri-urban environments.

Our preprocessing workflow prioritized geometric correction and spectral-spatial integrity through careful algorithm selection and parameter tuning^74^. For pan-sharpening, the High Pass Filter (HPF) algorithm was selected due to its ability to preserve spectral fidelity and edge sharpness^75^. While NNDiffuse, MIHS, and Subtractive Resolution Merge are more commonly used fusion methods, these alternatives often led to spectral degradation in our subsets^76,77^. HPF outperformed them in preserving classification-relevant features^78^, which was critical for accurate object delineation of fine-scale features such as roof structures and vegetation types. The SAM calculation, measures the spectral angle deviation between the fused HPF pan-sharpened bands and the original multispectral image, bilinearly resampled to match the pan-sharpened grid. The analysis yielded a mean SAM value of 1.52° (± 1.65°) and a median SAM value of 1.07°, values well within the range indicative of high spectral fidelity for pan-sharpened imagery^79^. These low angular errors indicate that upscaling the multispectral bands to the 0.31 m panchromatic resolution preserved the underlying multi-band radiometric profiles required for fine-scale classification. Isolated pixel-level spikes (maximum SAM = 78.50° were concentrated at sharp geometric boundaries and structural building shadows, where minor positional misalignment during resampling can locally inflate the spectral angle,these outliers had a negligible effect on the overall spectral fidelity of the dataset. As the original multispectral image was resampled prior to comparison, the reported SAM values reflect both pan-sharpening performance and a small degree of resampling-related smoothing, consistent with standard practice for this type of no-reference spectral fidelity check.

Another preprocessing step involved incorporating supplementary layers to improve object separability. While spectral indices like SAVI and NBEI have previously been used to enhance class separability in complex urban settings^80^, their combined use alongside Canny edge detection as primary segmentation inputs rather than post-processing tools represents a departure from conventional practice. In our study, SAVI was effective in normalizing soil variations across exposed patches and identifying them as bare ground, while NBEI improved the identification of roof types, distinguishing materials such as metal, slate, thatch, and unfinished roofs while suppressing vegetation and soil^43^. Together, these supplementary layers enhanced the delineation of settlement boundaries, roof types, vegetation types, and roof edges, outperforming the use of multispectral bands alone. The Canny Edge Detection layer, derived from combining eight spectral bands, improved segmentation quality. Edge detection algorithms such as the Canny detector have been widely used in remote sensing as a preprocessing step for feature extraction, enhancing object identification by detecting significant local intensity changes^44^. Incorporating the Sobel Edge Detector within the Canny computation further reduced point noise and enhanced smoothing, which was particularly important given the very high resolution of the imagery^81^.

Previous studies have demonstrated that structured classification enhances the representation of nested features and improves thematic consistency^82^^,^^83^. In this study, a Hierarchical Classification Scheme (HCS) was implemented to structure class relationships and reduce the segmentation search space, guided by ground truth data, which is widely recognized as essential for context-aware classification^7,71^. The HCS defines both vertical and horizontal nesting of classes and informs the assignment of layer weights across segmentation levels, as illustrated in Fig. 2. By aligning object classes with real-world landscape units and distributing them across four hierarchical segmentation levels, the approach enhances classification consistency and better captures the functional complexity of the peri-urban landscape. Consequently, the framework supports a more structured interpretation of spatial heterogeneity while reducing iterative trial-and-error in segmentation and classification. The corresponding layer weights used in the segmentation are provided in the appendix Table S2.

Segmentation and classification typically require iterative trial-and-error, particularly in heterogeneous environments^84^. Tools such as ESP2, which apply local variance and covariance methods^52^, were useful for identifying initial scale parameters and reduced trial-and-error iterations to only three, by providing optimal seed for the bottom-up approach. However, they were insufficient for accurate layer weighting and parameter tuning in highly variable contexts. ESP2 lacks flexibility in identifying parameters beyond scale for Multiresolution Segmentation (MRS), is limited to three segmentation levels, and performs better in homogeneous landscapes^85^. In heterogeneous settings, it often produces inconsistent results^86^. Therefore, manual fine-tuning of parameters was necessary to achieve segmentation quality consistent with the physical structure of the peri-urban landscape. Applying Multi-Threshold Segmentation (MTS) to the NBEI layer within Level 2 of the MRS framework further improved rooftop differentiation, allowing all major roof types to be identified^87^. The MRS approach, through the adaptive tuning of scale, shape, and compactness parameters, produced segmentation outputs that closely aligned with the physical structure of the peri-urban landscape. This is consistent with the findings of Tian and Chen^88^, who highlighted the limitations of relying solely on automated tools in diverse landscapes. Although five segmentation levels were initially planned, the fifth level was discarded due to overgeneralization and redundancy with level four, which already captured the relevant structural detail.

Feature selection remains one of the most complex and time-consuming steps in OBIA-based classification, given the large range of object-based features and the lack of systematic guidance for identifying optimal subsets in heterogeneous environments^7,56^. Existing approaches largely depend on manual trial-and-error, with limited structured frameworks for progressive feature reduction. To address this limitation, we propose a **c**oordinated, sequential feature selection framework that integrates multiple statistical methods at different stages of the selection process (Fig. 2). This framework moves beyond isolated feature ranking approaches by structuring feature reduction from general filtering to class-specific discrimination. Coefficient of Variation (CV) was first used to remove non-informative and unstable features, although its sensitivity to outliers is a known limitation^89^. Feature Space Optimisation (FSO) was then applied to quantify spectral distances between classes, although it proved insufficient for spectrally similar land use/cover categories. Linear Discriminant Analysis (LDA) was subsequently applied to selected class pairs with high spectral overlap, improving class separability despite its computational cost^90^. Finally, Otsu’s method^91^ was extended from binary to multi-threshold segmentation, enabling refined class separation when combined with auxiliary layers, with thresholds derived between the 5th and 95th percentile feature ranges. This integrated approach enhances feature filtering efficiency, improves class separability, and increases segmentation consistency in high-resolution heterogeneous landscapes.

The classification results reveal fine-scale LUC maps (Fig. 6) that exhibit distinct spatial patterns across the four study sites, closely corresponding to the peri-urban development trajectory proposed by Patric and Schaab^28^. This framework conceptualizes settlement evolution in Mankweng and its surroundings as a staged process, ranging from initial land allocation to more consolidated urban forms. Across the study sites, the observed spatial configurations reflect this progression. Early-stage areas are characterised by land allocation or Permission to Occupy (PTO), followed by the emergence of metal shack structures with limited infrastructure provision. Intermediate stages show increasing densification accompanied by partial service delivery, while later stages are marked by the coexistence of formal and informal structures. The most advanced stage corresponds to regularised settlements with established service connections. In addition to this general progression, two distinct spatial trajectories were identified. Planned expansion areas in Mankweng represent formally guided growth processes, while villagised settlements in Ramogale reflect a more traditional, culturally rooted settlement structure that diverges from the linear peri-urban development sequence. Together, these patterns highlight the spatial heterogeneity of peri-urbanisation, demonstrating that development does not follow a uniform pathway but instead reflects multiple coexisting trajectories shaped by planning interventions and socio-spatial context.

The thematic resolution achieved in this study advances peri-urban LUC mapping beyond what is typically provided by broader categorical or pixel-based approaches^92,93^. The resulting fine-grained classification supports three key applied contributions relevant to urban and peri-urban remote sensing and spatial planning in rapidly changing fragmented landscapes^94^. The differentiation of settlement structure types enables the spatial delineation of peri-urban development patterns at a level of detail not previously documented in former homeland contexts in sub-Saharan Africa. Second, spatial indicators such as roofing materials and bare ground extent function as indirect proxies for socio-economic conditions and livelihood strategies^95^, offering a remotely sensed basis for inferring wealth gradients and identifying service gaps without reliance on household survey data. Third, the classification captures the highly fragmented nature of peri-urbanisation in Mankweng characterised by the coexistence of formal and informal settlements, transitional land uses, and agricultural plots thereby revealing livelihood transitions and service inequalities that are typically obscured in coarser land cover products^28^. Beyond settlement typologies, several extracted landscape classes have direct planning relevance. Dumpsite detection informs infrastructure and public health planning^96^, erosion-prone area delineation highlights land degradation and the decline of subsistence agriculture, and sand mining zones reflect the expansion of informal livelihood activities^97^. The LUC maps across the four study sites reflect clear gradients of peri-urban transformation. Mankweng exhibits a highly structured settlement pattern with dense and well-established infrastructure, indicative of advanced consolidation. In contrast, Nobody represents transitional and early development phases, characterised by a heterogeneous mix of land uses, including the emergence of informal shack structures in former mashemos and partially completed buildings, particularly north of the main road. This pattern suggests active densification and rapid spatial expansion, further supported by the relatively high proportion of unfinished roofs (Fig. 7), which highlights uneven development pressures. Nchechane reflects a distinct early and irregular settlement morphology. Based on personal communication, the area developed prior to formal village planning, with initial land occupation occurring in an unstructured manner and parcel sizes shaped by household resources. Subsequent villagisation introduced formal boundaries that were superimposed onto pre-existing plots. As a result, spatial organisation remains uneven, with some households retaining larger parcels that are now partially used for subsistence agriculture, including maize cultivation and orchards. Ramogale corresponds closely to the villagisation model described by De Wet^98^. Betterment planning was introduced in the early 1980s (1980–1982), resulting in a more regularised layout with clearly defined parcel boundaries. However, the area still retains elements of traditional land use practices and exhibits a coexistence of formal and informal structures, distinguishing it from the more fully consolidated pattern observed in Mankweng. Overall, the LUC maps capture a clear continuum of peri-urban transformation across the study area, ranging from initial PTO-based land allocation in mashemos in Nobody to fully serviced and consolidated settlements in Mankweng. The OBIA-based classification effectively resolves this progression by distinguishing roof types, settlement configurations, and mixed land use patterns. These spatial indicators function as proxies for development stage, livelihood strategy, and governance history, consistent with findings from other rural–peri-urban transition contexts^99^, and highlight the strongly path-dependent and spatially heterogeneous nature of peri-urbanisation.

Recent studies have increasingly explored deep learning and machine learning approaches, including Convolutional Neural Networks (CNNs), You Only Look Once (YOLO), and the Segment Anything Model (SAM), for urban land cover extraction^100–103^. These approaches have demonstrated strong performance in structured urban environments where large, high-quality labelled RGB datasets are available. However, such datasets remain limited in many peri-urban regions of sub-Saharan Africa, particularly in fragmented and rapidly transforming landscapes characterised by heterogeneous settlement structures and mixed land uses.This constraint is not only practical but structural: CNN architectures require large numbers of labelled training samples to tune their many internal parameters, and the technical infrastructure, computational resources, and annotated satellite imagery needed to meet this requirement remain comparatively scarce across much of the African continent^104^. SAM, meanwhile, was trained predominantly on natural RGB imagery and exhibits a documented domain gap when applied to remote sensing data,its zero-shot segmentation performance degrades on multispectral inputs and lower-resolution imagery, both of which are common in peri-urban satellite datasets of the kind used in this study^105^. In this context, the OBIA-based framework presented in this study provides a practical alternative by integrating domain knowledge, multispectral information, and hierarchical contextual relationships while requiring relatively limited labelled training data. This enabled the delineation of complex peri-urban land use patterns that are often difficult to capture using purely pixel-based or data-intensive approaches. Nevertheless, the growing accessibility of deep learning models presents important opportunities for future research. Hybrid approaches combining OBIA with deep learning-based feature extraction or segmentation may further improve classification robustness, transferability, and automation in heterogeneous peri-urban environments. Future studies should therefore investigate the integration of OBIA and AI-driven methods using expanded training datasets and multi-temporal imagery across comparable peri-urban settings.

Evaluation of the classification results was conducted at two complementary levels. A visual assessment examined spatial coherence, thematic consistency, and the delineation of key classes such as settlement types and vegetation cover using the judgment-based scoring framework presented in Table 1. This approach prioritised contextual interpretability in data-scarce environments and provided a grounded qualitative evaluation of classification reliability. A quantitative accuracy assessment was performed using 471 field-collected ground truth points, resulting in an overall accuracy of 90.2% and an estimated Kappa coefficient of 0.81, indicating strong performance across the 38 LUC subclasses. However, the ground truth dataset used for validation also partially informed class training and ruleset development, which may have introduced optimistic bias into the reported accuracy estimates^65,66^. This limitation is well recognised in OBIA-based studies, particularly where fully independent validation datasets are unavailable^7^. A further limitation concerns the asymmetric validation coverage across the 38-subclass hierarchical scheme. The reported overall accuracy of 90.2% is derived exclusively from the 13 subclasses for which sufficient field-collected ground truth points were available,it does not statistically extend to the remaining 25 subclasses, which were assessed through visual scoring on a 1–10 scale. Even within these 13 quantitatively assessed subclasses, the density of reference points was limited, averaging approximately 13 points per class from a total of 470 field-collected points, reflecting the practical constraints of ground data collection across the fragmented and partially inaccessible peri-urban landscape.This asymmetry introduces two distinct caveats for interpreting the reported accuracy. First, because the overall accuracy figure is computed only from the well-sampled classes, it may carry an optimistic bias relative to the classification’s true performance across the full class scheme; overall accuracy estimates are known to be sensitive to which classes are represented in the reference sample and to the independence of that sample from the training data^106,107^. Second, since visual scoring reflects a qualitative judgment of spatial coherence and thematic plausibility rather than a quantifiable error estimate, localised misclassifications, boundary errors, and ruleset confusions among the 25 visually assessed subclasses remain unquantified. The 90.2% accuracy should therefore be read as representative of the dominant, well-sampled land cover classes, not as evidence of comparable reliability across the full 38-class scheme. The visually scored subclasses are best treated as exploratory spatial layers rather than fully validated quantitative products until independent ground truth data become available.

When contextualized against regional literature as in Sect. “Accuracy assessment”, these findings mark a meaningful shift from conventional mapping approaches toward higher-fidelity classification in sub-Saharan peri-urban environments. Previous studies in the Mankweng landscape have relied on coarser-resolution sensors and pixel-based classifiers, which limited their scope to broad, generalized categories^23,68,69^. While such approaches can achieve high accuracies for spectrally distinct features^68^, they struggle to resolve the fine spatial gradients and mixed land uses characteristic of rapidly transforming settlements, as reflected in the comparatively lower agreement reported by Vásquez Tavera et al.^69^. The higher classification agreement achieved in this study indicates that resolving fine-grained peri-urban structure requires aligning high-spatial-resolution imagery with object-based rather than pixel-based logic. By treating morphologically coherent image objects as the unit of analysis, rather than individual spectrally mixed pixels, the framework navigates the local heterogeneity that typically limits conventional classification workflows. This suggests that fine-grained thematic differentiation is achievable in data-scarce peri-urban settings without a substantial loss in classification reliability.

The transfer of the ruleset from the western to the eastern scene, requiring only minor adjustments to feature thresholds, provides an initial indication of methodological transferability under varying scene conditions. Nevertheless, future studies employing comparable imagery, class schemes, and validation strategies are still required to support rigorous cross-study benchmarking and methodological evaluation. The feature set identified for the western scene, was retained unchanged for the eastern scene; no additional or alternative features were required to distinguish classes. The adjustment involved recalibrating the Otsu-derived threshold values for the existing features to the eastern scene’s spectral distribution, rather than a re-identification of discriminative features. This distinction is relevant given that the two scenes differed in acquisition geometry, with a satellite azimuth difference of approximately 20° and a 4.6° difference in obliquity, corresponding to off-nadir viewing angles of approximately 22.7° and 27.3° for the western and eastern scenes respectively. These geometric and radiometric discrepancies were addressed upstream during orthorectification and pan-sharpening, prior to feature extraction and ruleset application. That threshold-only recalibration was sufficient to maintain classification performance after this preprocessing suggests that the selected feature set was reasonably robust to moderate differences in viewing geometry, though the extent of this robustness was not systematically tested. Concretely, the lower boundary threshold derived via Otsu’s multi-thresholding for the New Built-up Extraction Index (NBEI) had to be raised slightly in the eastern scene to consistently isolate metal and slate roof structures without omitting edges obscured by the altered shadow angles resulting from the different acquisition geometry. Similarly, the Soil Adjusted Vegetation Index (SAVI) threshold used to distinguish sparse bushland from low-albedo bare soil required a small downward shift to account for localised illumination intensity differences between the two scenes.

The transferability of the ruleset demonstrated here is subject to clear boundary conditions and should not be read as evidence of unconstrained transferability across seasons, sensors, or geographic regions. Temporally, transferring this ruleset to multi-seasonal imagery would likely introduce more substantial spectral shifts than those observed between the western and eastern scenes, driven by vegetation phenology (e.g. senescent versus actively growing vegetation) and changing soil moisture. The spectral index thresholds (SAVI, NBEI) would require recalibration or even a different selection of supplementary layers under these conditions, and, as observed in the west-to-east transfer described above, the geometric and textural rules, including the Canny edge layers used for building and road delineation, are also not fully insensitive to changes in acquisition conditions and would likely require re-tuning rather than being treated as fixed, depending on the use-case. Geographically, transferring the framework to other peri-urban regions is further constrained by regional variation in architectural styles (e.g. dominant roofing materials) and local vegetation composition^108^. In such cases, the hierarchical logic of the workflow and the automated feature-selection pipeline are expected to remain applicable in structure, but the specific classification thresholds and the Hierarchical Classification Scheme (HCS) itself would need to be manually adapted to the semantic and spectral context of the target region. Formal transferability across different landscape contexts, seasons, and image acquisition conditions therefore remains untested and is identified here as a priority for future work rather than a demonstrated property of the current framework.

A further limitation of this study is that the use of a single image acquisition cannot fully capture the dynamic and evolving nature of peri-urban landscapes, thereby limiting temporal insight into land use change and settlement transformation processes. Additional constraints arise from the reliance on manually developed rulesets and the limited sample representation of low-prevalence classes such as burnt areas, kraals, and dumpsites^7^. These factors may affect class generalisability and increase sensitivity to operator decisions during classification. Practical limitations are also associated with the resource-intensive nature of the workflow. The combination of multi-level segmentation, feature selection, and iterative ruleset refinement requires substantial processing time and computational capacity, which may constrain scalability across larger spatial extents without additional infrastructure investment. Furthermore, software-specific limitations may affect workflow portability and reproducibility across platforms. Despite these constraints, the framework relies primarily on commonly available statistical measures and widely accessible OBIA software environments, supporting broader applicability across diverse landscape settings beyond peri-urban regions in sub-Saharan Africa.

Future research should therefore prioritise multi-temporal image acquisitions to better capture seasonal and inter-annual land use dynamics, the development of semi-automated or adaptive ruleset generation approaches to reduce manual intervention, and expanded sampling strategies for underrepresented classes. Together, these improvements would enhance classification robustness, scalability, and transferability across heterogeneous peri-urban environments.

## Conclusion & outlook

This study demonstrated a transferable semi-automated OBIA-based classification approach specifically useful to the spatial complexity of peri-urban environments, particularly in semi-arid regions, using the Mankweng area as a case study. The workflow effectively captures fine-scale land use/cover and indirectly reveals the socio-economic patterns aligning settlement development stages as shown for four selected study sites. Additionally, incorporating known statistical measures such as, Coefficient of Variation (CV), Feature Space Optimization (FSO), Linear Discriminant Analysis (LDA), and Otsu’s multi-thresholding into the classification workflow ensures, minimal trial and error space and a structure to the process of OBIA commonly followed. Each of the selected statistical measures is applied beyond its binary usage in image processing and object identification. The workflow can also contribute to identifying a high number of fine resolution classes for advanced spatial analysis and deeper insights into other peri-urban contexts. While no new algorithms were introduced, the coordinated application of these statistical measures and segmentation strategies contributes a new approach to OBIA-based classification, reducing computational load and the extent of trial-and-error typically required.

Our OBIA-derived objects provide a basis for quantifying spatial structures and revealing dependencies of class relationships^109^. For further using the results of classification useful on the ground, incorporating landscape metrics could enhance interpretation of spatial patterns at the landscape level^110^. These metrics can support understanding the settlement development stages, vegetation diversity, and land use mix, offering valuable indicators for spatial planning interventions and policy formulation. Although feasible, the new OBIA-based approach could be also applied to other very high-resolution imagery such as acquired by unmanned aerial vehicles (UAV) although other, practical difficulties may arise. When applied to high-resolution datasets, such as Sentinel-2 which is free, broader spatial extents could be covered, however, the same fine-granularity of classes cannot be achieved. Either way, the framework can be applied to any other area of the globally ever-expanding peri-urban regions thus adding to a better understanding of their incredible diversity.

## Supplementary Information


Supplementary Information.



Supplementary Information.


## Data Availability

The data supporting the findings of this study are included within the manuscript and its supplementary information files. Additional datasets generated during the current study are available from the corresponding author upon reasonable request. The ALOS PALSAR dataset used in this study is publicly accessible at: https://www.earthdata.nasa.gov/data/projects/alos-palsar-rtc-project. The scripts used are deposited in a Zenodo repository https://doi.org/10.5281/zenodo.21328261 and are freely available for reuse.
