# Supplementary material for: Semi-automated object-based image analysis for peri-urban land use/cover classification using WorldView-3 imagery of Mankweng, South Africa
Source: Sci Rep. 2026 Aug 28;16:27075. doi: 10.1038/s41598-026-66042-x (PMC13524936; doi:10.1038/s41598-026-66042-x)
Supplement: Supplementary file 1 [file 41598_2026_66042_MOESM1_ESM.docx]

**Table S1**: The supplementary version of Table 1 adds a definition per subclass as well as the features used in ruleset development per class/subclass.

| **ID** | Class | Definition | Level of Segmentation | Number of Samples | Features | Classification Evaluation  (1 to 10) | Subset Values REUsed |
| --- | --- | --- | --- | --- | --- | --- | --- |
| 1 | Bare ground | - | 4 | 124 | GLCM Homogeneity (NIR2), GLCM Mean Red, HSI Hue, Mean SAVI, Ratio (NIR2), Density | 8 | Y |
| 1.1 | Bare ground ibs | Bare ground in a broad sense are areas devoid of vegetation cover, typically characterised by exposed soil. | 3 | 145 | Existence of Bare ground, Distance to roof types (metal, unfinished, slate, thatched) | 8 | Y |
| 1.2 | Sports ground | Large recreational ground adjacent schools or educational instituitions. Identified by proximity to the schools or educational buildings | 3 | 16 | Existence of Bare ground, Area | 8 | Y |
| 1.3 | Bare ground ins | Moderately bare ground surface, showing less severe disturbance compared to ibs. Closer to the class building. | 3 | 132 | Existence of Bare ground, Border to roof types (metal, unfinished, slate, thatched) | 7 | Y |
| 1.4 | Dry river bed | Seasonal or intermittent channels where water flows during periods of rainfall but are dry for most of the year. Follows an irregular linear pattern with exposed rocks. | 3 | 12 | Ratio rededge, Asymmetry, Ratio NIR2, Ratio NIR1, HSV Hue, Density, GLCM Homogeneity (NIR2), Mean Green, Brightness | 7 | Y |
| 1.5 | Outcrop | Exposed rock formations or geological features visible in the imagery with a distinct reflectance. | 3 | 77 | Ratio rededge, Ratio NIR1, Max diff, Ratio NIR2, Ratio blue, Density, Elliptic fit, Brightness, Rectangular fit, Mean NIR2, Mean NIR1 | 8 | Y |
| 1.6 | Erosion | Areas showing signs of soil erosion, such as gullies, rills, or sediment deposition. Associated within maize fields or non-settled areas. | 3 | 9 | Existence of Bare ground, Mean SAVI, Brightness | 6 | N |
| 1.7 | Dumpsite | Areas used for waste disposal, typically characterised by accumulated trash or landfill activities. Has a specific reflectance to the area. | 3 | 19 | Max difference, Shape index, Brightness, Mean green, GLCM Homogeneity (NIR1), Ratio (NIR2), Mean yellow, Border contrast NIR2, Border contrast NBEI | 6 | Y |
| 1.8 | Kraal | Enclosures or pens for livestock, commonly found in agricultural landscapes. | 2 | 7 | Mean SAVI, Shape Index, Border to roof types (metal, unfinished, slate, thatched) | 6 | Y |
| 1.9 | Mining | Areas where extraction of minerals or ores occurs, characterised by disturbed terrain and infrastructure associated with mining operations. Associated within maize fields or non settled areas. | 3 | 11 | Existence of Bare ground ibs, Mean SAVI | 8 | N |
| 2 | Water | - | 4 | 4 | Mean SAVI, Mean NIR2 | 10 | Y |
| 2.1 | Open water | Areas containing water. Contains all water bodies natural and manmade. In this landscape also sewage and water treatment plants. | 4 | 12 | Mean SAVI, Mean NIR2, Area | 9 | Y |
| 2.2 | Swimming pool | Artificial pools | 4 | 2 | Area, Mean SAVI, Mean NIR2 | 7 | Y |
| 2.3 | River | Linear waterbodies | 4 | 9 | Length, Mean SAVI, Mean NIR2 | 8 | Y |
| 3 | Vegetation | Vegetated areas | 4 | 9 | GLCM Homogeneity (Red Edge), Ratio rededge, GLCM Homogeneity (NIR2), Ratio (Blue) | 9 | Y |
| 3.1 | Maize | Fields or areas where maize, the most commonly cultivated food crop in the study area, is grown. It can be identified from its distinctive texture and shape when viewed from the satellite imagery. | 3 | 198 | Distance to roof types (metal, unfinished, slate, thatched), Mean NIR2, Rectangular fit, Mean NIR1, Brightness, GLCM Homogeneity (Red Edge), GLCM Homogeneity (NIR2), Ratio (Blue), max pixel value rededge | 7 | Y |
| 3.2 | Mashemo | Communal farmlands in the study area, mostly used for maize cultivation. Distinguished by the area of maize cultivation and rectangular plot shape | 3 | 17 | Area Maize, Distance to roof types (metal, unfinished, slate, thatched) | 7 | Y |
| 3.3 | Fallow | Agricultural land left uncultivated for a season or more, exhibits a typical dark shade in the soil within agricultural areas or proximity to settled areas. | 3 | 106 | Existence of Grassland, Ratio rededge, Ratio blue, Max pixel value NIR2, Rectangular fit, GLCM Homogeneity (Red Edge), GLCM Homogeneity (NIR1), GLCM Homogeneity (NIR2), Max pixel value NIR1, Max pixel value red edge, Distance to roof types (metal, unfinished, slate, thatched) | 6 | Y |
| 3.4 | Bushland/  Shrubland | Areas dominated by shrubs or small trees, often found in semi-arid regions or on marginal lands of fields and roads. | 3 | 134 | Ratio rededge, Distance to roof types (metal, unfinished, slate, thatched), Ratio NIR2, Density, GLCM Homogeneity (Red Edge), GLCM Homogeneity (NIR1), GLCM Homogeneity (NIR2), Ratio NIR1 | 6 | N |
| 3.5 | Grassland/  Savannah | Open landscapes dominated by grasses with presence scattered trees or shrubs. | 3 | 122 | Ratio rededge, GLCM Homogeneity (NIR1), Ratio blue, Max pixel value NIR2, Not bordering rooft ypes (metal, unfinished, slate, thatched) | 6 | N |
| 3.6 | Burnt area | Land that has undergone burning, characterised by charred vegetation and distinct blackened soil. | 3 | 5 | Ratio rededge, GLCM Homogeneity (NIR1), GLCM Homogeneity (NIR2), Ratio (Blue), Ratio NIR1, Max pixel value coastal, Brightness,  GLCM Homogeneity Yellow, Mean rededge, Rectangular fit | 3 | N |
| 3.7 | Riverine vegetation | Vegetation found along riverbank, dry river beds and water, often characterised by dense growth due to the availability of water. Characterised by bushes, burnt vegetation, fallow and grassland in this landscape. | 3 | 34 | Border to Bushland, Border to Burnt area, Border to Grassland, Border to Water | 7 | N |
| 3.8.1 | Trees | Individual tree cover within the landscape, distinguished from other vegetation types by their height/shadow and canopy shape. | NBEI_MTS (2) | 112 | Mean NBEI | 7 | Y |
| 3.8.2 | Single tree | Isolated trees scattered within the landscape, often found in agricultural or urban areas. | NBEI_MTS (2) | 123 | Mean NBEI, Area | 7 | Y |
| 3.8.2 | Fruit tree | - | NBEI_MTS (2) | 25 | Mean NBEI, Distance to house roof types (metal, unfinished, slate, thatched) | 7 | Y |
| 3.8.3 | Tree stand | Groupings or clusters of trees within the landscape forming distinct patches of vegetation. Identified by the area of the canopy. | NBEI_MTS (2) | 78 | Mean NBEI | 7 | Y |
| 3.9 | Aloe | Areas dominated by Aloe species, often found in arid or semi-arid regions. | 2 | 46 | Existence of grassland, Existence of bushland, Mean NBEI, Area | 3 | N |
| 4 | Built-up & infrastructure |  |  |  |  |  |  |
| 4.1 | Tarmac road | Paved roads or highways constructed using asphalt or concrete. | 4 | 13 | Length/Width, Rectangular Fit, Density  GLCM Homogeneity (NIR2), HSI Hue, Ratio (NIR2), Shape Index | 8 | Y |
| 4.2.1 | Metal roof | Different types of roofing materials and styles used in building construction, such as metal, tile/slate, unfinished or thatch roofs. | NBEI_MTS (2) | 167 | Mean NBEI | 8 | Y |
| 4.2.2 | Slate roof |  |  | 167 | Mean NBEI | 8 | Y |
| 4.2.3 | Thatched roof |  |  | 12 | Mean NBEI | 7 | Y |
| 4.2.4 | Unfinished roof |  |  | 112 | Mean NBEI | 6 | Y |
| 4.3 | Commercial buildings | Areas associated with manufacturing, processing, or industrial activities, typically characterised by factories, smokestacks, or industrial infrastructure. Larger buildings are categorised as industries. | 3 | 3 | Area, Asymmetry, Distance to tarmac road | 8 | Y |
| 4.4 | School | Educational institutions such as schools, colleges, or universities, often identifiable by campus buildings and proximity to the bare ground ibs. | 3 | 3 | Distance to sportsground, Existence of Industry | 8 | Y |
| 4.5 | Sheds | Small structures used for storage or shelter, typically found in agricultural or industrial settings. | NBEI_MTS (2) | 77 | Area-house  Distance to house | 6 | Y |
| 4.6 | RDP house | Subsidized housing in South Africa. Characterized by metal roof type with ridges and size of the area. | NBEI_MTS (2) | 68 | Existence of metal roof, Area | 6 | Y |
| 4.7 | Wall/Fence | Barriers or partitions constructed to enclose or separate properties, often made of concrete, brick, wood, or wire. | 2 | 109 | Length/width | 4 | N |
| 4.8 | Footpath | Trails or paths used by pedestrians. Narrow in comparison to the dirt roads. | 2 | 33 | Length/width  Shape index | 5 | N |
| 4.9 | Dirt road | Unpaved roads or tracks made of natural materials such as soil, gravel, or sand. Linear shaped and narrower in comparison to tarmac roads. | 3 | 79 | Length, GLCM Mean Red, Max Difference, Rectangular Fit, No border to roof types, Ratio red edge, Ratio blue, Ratio NIR1, Mean NIR2, Mean NIR1, Max pixel value NIR2 | 8 | Y |
| 4.10 | Water tank closed | Closed containers or reservoirs for storing water, often used for domestic or industrial purposes. A distinct circular shape associates with a water tank and can also be related with the height. | NBEI_MTS (2) | 24 | Mean NBEI | 8 | Y |
| 4.11 | Cemetery | Burial grounds or graveyards, often characterized by rows of tombstones or grave markers. | 3 | 41 | Elliptic Fit, HSI Hue, Max Difference, GLCM Mean (Red), Mean red edge, HSV Hue, Rectangular fit | 8 | Y |
| 4.12 | Carpark | Areas designated for parking vehicles, including parking lots, garages, or parking structures. Identified with proximity to the tarmac roads and commercial buildings. | 3 | 13 | Area, Border to tarmac road | 7 | Y |
