# Supplementary material for: Semi-automated object-based image analysis for peri-urban land use/cover classification using WorldView-3 imagery of Mankweng, South Africa
Source: Sci Rep. 2026 Aug 28;16:27075. doi: 10.1038/s41598-026-66042-x (PMC13524936; doi:10.1038/s41598-026-66042-x)
Supplement: Supplementary file 2 [file 41598_2026_66042_MOESM2_ESM.docx]

**Table S2:** Layer weights assigned per segmentation level in MRS. Weight order follows W, E, N, S as used in Figure 2. Higher value = higher weight. To maintain balanced layer contribution and allow comparison across segmentation levels, all weights were rescaled to a relative total of 10 per level. MS weight of 0.625 is applied equally across all 8 multispectral bands, contributing a combined total of 5.0 per level (4.0 at Level 1).

| **Level** | **W (MS)** | **E (Edge)** | **N (NBEI)** | **S (SAVI)** | **Rationale** |
| --- | --- | --- | --- | --- | --- |
| ***Level 5*** | 625 | 1 | 2 | 2 | Distinguish vegetation/non-vegetation and built-up/non-built-up areas |
| ***Level 4*** | 625 | 1 | 2 | 2 | Emphasise building boundaries and bare ground delineation |
| ***Level 3*** | 625 | 2 | 1 | 2 | Edge improves structural delineation; SAVI contrasts building structures |
| ***Level 2*** | 625 | 2 | 1 | 2 | NBEI  and SAVI combination delineates roof types and edges |
| ***Level 1*** | 0.5 | 3 | 1 | 2 | Edge prioritised for fine structural boundaries and diner class differentiation |

*W = WorldView-3 multispectral bands (8 bands); E = Canny Edge Detection layer; N = New Built-up Extraction Index (NBEI); S = Soil Adjusted Vegetation Index (SAVI).*
